# Recent parallel speciation in *Antirrhinum* involved complex haplotypes and multiple adaptive characters

**DOI:** 10.1111/mec.17101

**Published:** 2023-08-21

**Authors:** Matthew Barnbrook, Mario Durán‐Castillo, Jo Critchley, Yvette Wilson, Alex Twyford, Andrew Hudson

**Affiliations:** ^1^ University of Edinburgh School of Biological Sciences Edinburgh UK; ^2^ Royal Botanic Garden Edinburgh Edinburgh UK

**Keywords:** adaptation, *Antirrhinum*, gene flow, introgression, RADseq, snapdragon, speciation

## Abstract

A role of ecological adaptation in speciation can be obscured by stochastic processes and differences that species accumulate after genetic isolation. One way to identify adaptive characters and their underlying genes is to study cases of speciation involving parallel adaptations. Recently resolved phylogenies reveal that alpine morphology has evolved in parallel in the genus *Antirrhinum* (snapdragons): first in an early split of an alpine from a lowland lineage and, more recently, from within the lowland lineage to produce closely related sympatric species with contrasting alpine and lowland forms. Here, we find that two of these later diverged sympatric species are differentiated by only around 2% of nuclear loci. Though showing evidence of recent gene flow, the species remain distinct for a suite of morphological characters typical of earlier‐diverged alpine or lowland lineages and their morphologies correlate with features of the local landscape, as expected of ecological adaptations. Morphological differences between the two species involve multiple, unlinked genes so that parental character combinations are readily broken up by recombination in hybrids. We detect little evidence for post‐pollination barriers to gene flow or recombination, suggesting that genetic isolation related to ecological adaptation is important in maintaining character combinations and might have contributed to parallel speciation. We also find evidence that genes involved in the earlier alpine‐lowland split were reused in parallel evolution of alpine species, consistent with introgressive hybridisation, and speculate that many non‐ecological barriers to gene flow might have been purged during the process.

## 1 INTRODUCTION

Parallel evolution has provided important insights into the mechanisms of adaptation (Arendt & Reznick, 2008; Blount et al., 2018; James et al., 2023; Stern & Orgogozo, 2009) and has helped to identify adaptive characters and the underlying mutations and selective agents (e.g. Konečná et al., 2021; Rennison et al., 2019). In some cases, the parallel evolution of ecological adaptations has been proposed to lead to reproductive isolation, resulting in pairs of sister species, or incipient species, that have diverged for similar adaptations (e.g. Meier et al., 2018; Nosil et al., 2004; Wilson et al., 2007).

The best understood examples of parallel evolution (which we do not distinguish here from convergence for the reasons discussed by Arendt & Reznick, 2008) have been those around the species level that have a genetically simple basis, for example the evolution of insects resistant to the cardenolide toxins in their host plants (Zhen et al., 2012). However, genome‐wide studies suggest that parallel evolution of genetically complex adaptations is common (discussed by Lee & Coop, 2019). Reuse of existing mutations also occurs frequently in taxa that have access to the same alleles from standing ancestral variation or through hybridisation (Bohutínská et al., 2021; Conte et al., 2012), though this view may be biased because larger‐effect mutations are more likely to be detected and to be reused (Dittmar et al., 2016).

In plants, hybridisation between species is relatively frequent (Whitney et al., 2010) and can promote rapid homoploid speciation by providing new character combinations in hybrids (e.g. Rieseberg et al., 2003). Introgressive hybridisation between species is also well documented for flowering plants. For example, it can result in the capture of one parent's plastid genome by a nuclear genome largely from another lineage (e.g. Folk et al., 2017) or replacement of nuclear alleles at multiple loci involved in an ecological adaptation (e.g. Konečná et al., 2021; Le Corre et al., 2020; Rieseberg et al., 2003; and reviewed by Suarez‐Gonzalez et al., 2018). This raises the possibility that an ecological adaptation that contributed to the reproductive isolation of one pair of sister species may promote the parallel speciation of another pair after being transferred by hybridisation. However, an adaptation that involves multiple genes risks being recombined into maladapted genotypes in hybrids, which can explain why adaptive alleles are often found coupled with other barriers to hybridisation or recombination (Barton & De Cara, 2009; Fishman et al., 2013; Flaxman et al., 2014). Such barriers include chromosome rearrangements that can suppress both recombination and hybrid fitness (e.g. Rieseberg et al., 1999). Some of these barriers—those that reduce the formation or fitness of hybrids—may limit the potential for parallel evolution by introgression of a complex adaptive haplotype into another genome. Therefore, the genetic architecture of an adaptation and the factors that limit it being broken up by hybridisation and recombination are relevant to understanding how it can evolve in parallel. This is particularly relevant to the potential of hybridisation to promote parallel ecological speciation because the mechanisms isolating the donor of the adaptation are expected to restrict introgressive hybridisation into another lineage.

**FIGURE 1:**
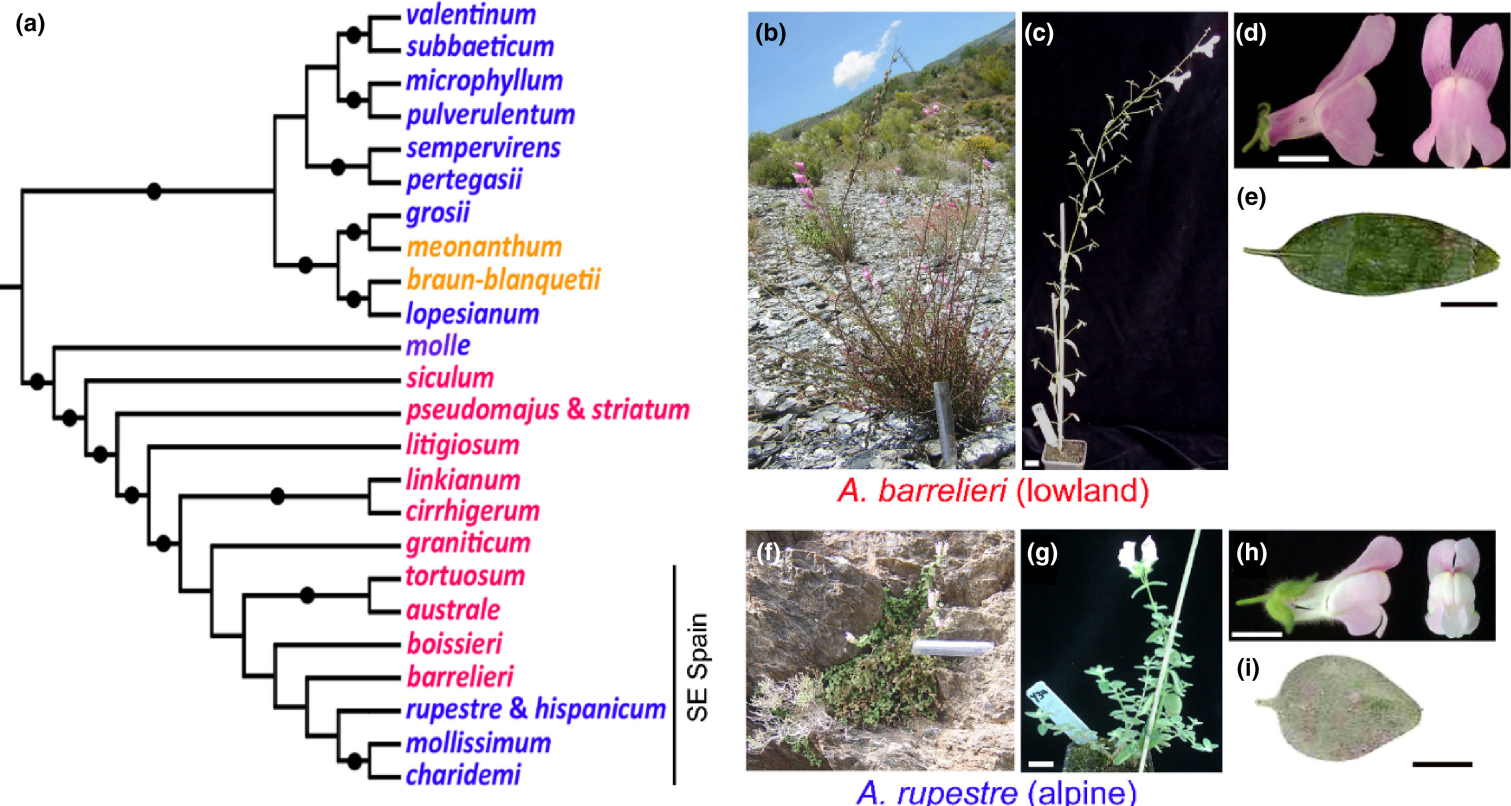
Parallel evolution of alpine morphology and *A. barrelieri* and *A. rupestre* phenotypes. (a) A cladogram showing assumed relationships between *Antirrhinum* species, based on Duran‐Castillo et al. (2021). Species with alpine morphology (taxonomic section *Kickxiella*) are in blue and taxonomic section *Antirrhinum* with lowland morphology in red. The two species in orange correspond to section *Streptosepalum* and are suggested to have evolved lowland morphology in parallel to the other lowland species. Where two species are unresolved they are shown on the same leaf. Supported nodes are marked with dots. (b) A representative of *A. barrelieri* (lowland morphology) in the field and (c–e) its glasshouse‐grown progeny. (f) *A. rupestre* (alpine morphology) in the field and (g–i) its glasshouse‐grown progeny. The same 150 mm ruler is shown in (b) and (f). Scale bars are 20 mm in (c) and (g) and 10 mm in (d, e) and (h, i).

The roles of genetically complex adaptations and hybridisation in parallel speciation are ideally tested in a study system in which inter‐fertile pairs of species have diverged for adaptive differences determined by multiple underlying genes. Parallel ecological speciation involving a suite of genetically complex characters has been suggested for the genus *Antirrhinum* (snapdragons). The genus has generated around 27 recognized species or subspecies within the last ~5 million years (Otero et al., 2021). Its species represent two ecomorphs—one, which we term alpine, is that of the 13 species traditionally placed in taxonomic section *Kickxiella* and found in rock‐faces, often at high elevation. The other ecomorph (‘lowland’) corresponds to the taxonomic sections *Antirrhinum* and *Streptosepalum*, containing 14 species that generally have wider ranges at lower elevations (Rothmaler, 1956; Webb, 2010). Phylogenies reconstructed from multiple nuclear loci support the early split of an alpine from a lowland lineage (Figure 1a; Duran‐Castillo et al., 2021; Otero et al., 2021; Wilson & Hudson, 2011). The alpine lineage then gave rise to species that are each endemic to a particular mountain range in Iberia (‘ancestral alpines’). These share with each other a suite of characters that includes being short, prostrate and highly branched with small hairy leaves and small, usually white flowers with magenta markings. The lowland lineage, in contrast, produced larger, more upright species, with larger, glabrous and narrower‐shaped leaves, and flowers that are predominantly magenta or yellow (Duran‐Castillo et al., 2021; Güemes, 2009; Rothmaler, 1956; Sutton, 1988; Webb, 2010; Whibley et al., 2006; Wilson & Hudson, 2011). The species with lowland morphology are each geographically more widespread in Iberia or elsewhere around the Mediterranean region (Rothmaler, 1956; Sutton, 1988). Importantly, alpine morphology appears to have evolved a second time, from within the lowland lineage in the Betic mountain system of southeast Spain (Figure 1a; Duran‐Castillo et al., 2021; Otero et al., 2021; Wilson & Hudson, 2011). This parallel divergence has led to young, closely related taxa in geographic proximity that represent contrasting alpine and lowland forms.

Morphometric studies have supported the classical taxonomic view that alpine and lowland ecomorphs of *Antirrhinum* differ for multiple characters (Wilson & Hudson, 2011). Crossing pairs of distantly related species with contrasting morphologies has further shown that most diverged characters are genetically complex (Costa et al., 2012; Feng et al., 2009; Langlade et al., 2005). For example, at least nine loci contribute to differences between the large, narrow leaves of *A. majus* (lowland) and the small, rounder leaves of *A. charidemi* (alpine; Langlade et al., 2005). However, these studies only identified loci for which the parents differ and so could not distinguish genetic and morphological differences that contributed to the initial alpine‐lowland divergence from those that accumulated subsequently. Most comparisons also used an *A. majus* cultivar as one of the parents and might therefore have included characters and genes selected during in its domestication. The species from southeast Spain, in contrast, provide the opportunity to examine the characters and genes involved in a recent alpine‐lowland divergence and to compare them to those of the earlier event.

The extent to which parallel evolution of alpine *Antirrhinum* species in southeast Spain involved new mutations or reuse of existing alleles is currently unclear. Reuse of mutations via introgression is a possibility because all *Antirrhinum* species are able to form fertile hybrids with each other when experimentally cross pollinated (Stubbe, 1966). Furthermore, the alpine taxa from southeast Spain carry a nuclear genome predominantly from the lowland lineage but the same derived plastid haplotype and alleles of a gene responsible for their hairy leaves with the ancestral alpine lineage (Jiménez et al., 2005; Liberal et al., 2014; Tan et al., 2020; Wilson & Hudson, 2011). This could be explained by introgression of existing alpine mutations and the plastid into the nuclear genetic background of the lowland lineage or by parallel assembly of the alpine plastid and hairy allele combination from ancestral polymorphism. Sequencing of genome fractions, using GBS or RADseq, failed to detect a significant excess of derived nuclear alleles shared between the independently evolved groups of alpines (Duran‐Castillo et al., 2021; Otero et al., 2021), suggesting that if introgression were involved, it must have involved genes that were mostly not sampled in the sequenced genome fractions.

Because they appear to have diverged for alpine and lowland morphologies in parallel to the earlier split, the southeast Spanish taxa may provide information about the genes and processes involved in parallel speciation. However, their taxonomy and relationships are unclear (Rothmaler, 1956; Sutton, 1988). With the exception of the geographically isolated *A. charidemi*, all have been reported to show similar levels of morphological variation within and between species (Wilson & Hudson, 2011), which may be the result of recent gene flow between them, incomplete fixation of ancestral polymorphisms or both. Although problematic for delimitation of species, this variation could provide a further opportunity to understand how different combinations of characters and genes contribute to alpine or lowland adaptation and to identify factors that could prevent genetically complex adaptations being broken down by hybridisation and recombination in these sympatric species.

Here, we examine the *Antirrhinum* species from the Alpujarra region, on the southern flank of the Sierra Nevada in southeast Spain, focussing on two endemic species that represent contrasting alpine and lowland forms and overlap almost entirely in range. We find that they are very similar genetically and show evidence of recent hybridization but are distinct for a suite of heritable morphological characters that correlate with the habitats in which they are found, as expected for ecological adaptations. Experimental hybrids between the species indicate that almost all their morphological differences are under the control of multiple, unlinked genes and that relatively few of these genes affect more than one character, so that parental characters become mismatched in hybrids. We use RADseq of the species and of an experimental hybrid population to test for mechanisms that may keep parental character combinations intact, but find little or no evidence for clustering of divergent genes or of loci that underlie morphological differences, for suppression of recombination in hybrids or for genetic incompatibilities that may act as barriers to gene flow. Comparative mapping of genes underlying species differences in morphology suggests that some of the alleles involved in the original alpine‐lowland split were reused in the later parallel divergence in southeast Spain.

## 2 MATERIALS AND METHODS

### 2.1 Plants: taxonomy, culture and phenotypes

The taxonomy of *Antirrhinum* in southeast Spain has been inconsistent. We follow that of Rothmaler (1956), who carried out the most extensive sampling of the region, and base our identification on resampling of populations at locations from which he collected and described *A. hispanicum* (Alhama), *A. rupestre* (Trévalez, Pampaneira), *A. mollissimum* (Enix), *A. barrelieri* (Mulhacén, Bobadilla) and *A. tortuosum* (El Torcal de Antequerra). By precedent, the name *A. barrelieri* properly belongs to a different taxon, from northeast Spain (Ferrer‐Gallego & Güemes, 2020). Consequently, the name *A. controversum* is sometimes used for Rothmaler's more southern taxon (Sutton, 1988), though we retain the name *A. barrelieri* here for consistency. In sampling within the Alpujarra region, we did not select populations based on morphology—for example, we did not reject any samples with morphologies intermediate between Rothmaler's type populations—to avoid sampling bias affecting studies of character distributions.

Plants grown from wild‐collected seeds and experimental hybrids were grown for phenotype analysis in a greenhouse at Edinburgh between March and September at 19–25°C. Seeds were sown into John Innes (JI) No 1 compost (Evergreen Garden Care Ltd) and transplanted to 60 mm × 60 mm × 80 mm pots of JI No 2 compost once their cotyledons had expanded fully. After around 6 weeks, plants were transferred to 100 mm × 100 mm × 110 mm pots of JI No 2 compost. Light intensity was maintained at a minimum of 480 μmol m^−2^ s^−1^ during 16 h days by supplementing natural light with metal halide lamps. All seedlings that germinated over a 4‐week period were transplanted, to minimize selection against more dormant genotypes.

To generate an F_2_ mapping population, an individual of *A. barrelieri* from a population near Alcolea, Almería, was crossed as female to *A. rupestre* from Capileira, Granada. As F_1_ progeny were self‐incompatible, the same two full‐sib F_1_ individuals were crossed to each other reciprocally, as pollen parent in one direction of the cross and seed parent in the opposite direction, to generate two F_2_ sub‐populations of 115 plants, selected at random from the transplanted seedlings available. No significant difference was detected for character means or variances between the F_2_ progeny from opposite directions of the F_1_ inter‐cross; the lowest *p*‐value from *t*‐tests comparing the two sub‐populations for each of the 11 characters was .0524 and the lowest *p*‐value from *F*‐tests for equal variances .01813, compared to a Bonferroni‐corrected *α* for significance at the .05 level of .0046. Therefore, both F_2_ sub‐populations were pooled for further analysis.

We measured morphological characters of *A. barrelieri* (*n* = 88) and *A. rupestre* (*n* = 64) in the field and grew progeny from open‐pollinated seed in a glasshouse (a total of 154 *A. rupestre* and 105 *A. barrelieri* progeny; the distribution of samples across populations is given in Table S1). Because both species are self‐incompatible, the pollen parents of wild collected seeds were not known, though assumed to be different to the seed parent. Glasshouse grown plants were measured once five flowers had opened: plant height (PlHt) from the cotyledons to the first flower, internode length (IntL) as the average for the basal 10 internodes and branching index (BrIn) as the ratio of the number of vegetative nodes in the longest lateral branch to the number in the primary axis. Flower number (FlN) was a count of the total number of flowers produced in the primary inflorescence. One leaf at metamer 4 (where cotyledons are part of metamer 1) was excised, flattened and scanned. Leaf area (LfA), and maximum leaf length (without the petiole) and maximum width were estimated from the image and used to calculate the length: width ratio (LfLW). The length of one dorsal sepal (SepL) was measured in the same way. A single flower from each plant were excised, photographed from the side and ImageJ used to estimate pedicel length (PedL), dorsal petal angle (PetAn; the obtuse angle formed between the dorsal petals and the proximal‐distal axis of the corolla tube) and flower length (FlL; from the pedicel‐calyx junction to the corolla palate). Dorsal petal length (PetL) was measured from images of the excised and flattened dorsal corolla as the mean distance between the distal tips of the corolla lobes to the midpoint of their junction with the tube. Presence or absence of trichomes was assessed visually on vegetative stems and leaf blades of metamer 5. Plants in the field were recorded in the same way, except that they were not all at the same developmental stage as each other nor their glasshouse‐grown progeny, and therefore a mature leaf judged typical was used if a metamer 4 leaf was not available. Sample sizes are given in Table S1 for plants in the field. For both field and glasshouse plants, the values for four characters (PlHt, BrIn, LfA, NFl) were natural log‐transformed to make them more normally distributed. Characters in F_1_ and F_2_ hybrid progeny were measured in the glasshouse, as for offspring of *A. barrelieri* and *A. rupestre*.

### 2.2 Population genetics

To examine chloroplast haplotypes carried by the five sampled species, DNA was extracted from frozen glasshouse‐grown or dried field‐collected tissue and two chloroplast loci (*trnD*‐*trnT* and *trnS*‐*trnR*) amplified from a subset of each species, as described previously (Wilson & Hudson, 2011). All yielded identical sequences at each of the loci (GenBank accessions FR690437 and FR690213). They shared an *Mse* I restriction site in *trnD*‐*trnT* that is characteristic of the derived haplotype of ancestral alpine section *Kickxiella* and lacked a *Tfi* I restriction site in *trnS*‐*trnR* found mainly in lowland section *Antirrhinum*. Restriction digests confirmed that only this haplotype was present in all samples (8–10 individuals from each of 24 populations).

To examine genetic delimitation of the southeast Spanish species and to relate this to their morphologies, we AFLP genotyped wild accessions (72 accessions of *A. barrelieri* from 10 populations, 49 *A. rupestre* from 8 populations, 29 *A. hispanicum* from 4 populations, 9 *A. mollissimum* from two populations and 10 *A. tortuosum* from a single population). AFLP were detected by selective amplification of *Pst* I‐*Mse* I restriction fragments and markers that could not be recovered in all replicates of the same plant or that showed potential size homoplasy removed as described previously (Wilson & Hudson, 2011). The remaining 506 polymorphic loci were scored as dominant markers and the statistical software package PAST (Hammer et al., 2001) used for genotype PCA with Jacquard distances. Markers showing F_*ST*_ values between *A. barrelieri* and *A. rupestre* that were significantly higher than expected for random distribution of alleles between the two species were identified with Mcheza (Antao & Beaumont, 2011). Structure assignment (Falush et al., 2007) used AFLP genotypes and models allowing admixture and inference of allele frequencies. We examined assignment of individuals to one of either two genetic clusters (*K* = 2), to test whether clusters correspond to alpine and lowland morphologies, or to five clusters (*K* = 5), corresponding to the number of recognized species, which also proved to be the value of *K* best fitting the data using the Δ*K* method of Evanno et al. (2005). We examined a wide range of parameter values, testing each with eight replicate runs (each with 20,000 burn‐in and 120,000 experimental states) and found the best fit to data over *K* = 1–5 was with *α* = 1, *λ* = 1, population‐specific *α* = 0, *MIGPRIOR* = 0.001, independent alleles, an *FPRIOR* mean of 0.10 and standard deviation of 0.10. Assignment was done without reference to taxonomy or collection sites (i.e. *LOCPRIORS* were not used). Congruence of replicates was assessed with CLUMPP (Jakobsson & Rosenberg, 2007) and assignments plotted with DISTRUCT (Rosenberg, 2004) and Google Earth.

To test whether there was a relationship between the degree of admixture in *A. barrelieri* populations and their physical proximity to *A. rupestre*, and vice versa, we used the populations means for the proportion of the genomes of *A. barrelieri* assigned to the ‘alpine’ ancestor, or to ‘lowland’ ancestry in *A. rupestre* populations, by Structure at *K* = 2. We regressed the admixture value for each population onto the distance, in kilometres, to the nearest population of the other species (the distance was set to 0.1 km for the sympatric rupS/barS mixed population). We also tested whether there was a relationship between intermediate morphology and proximity to the other species in an analogous way. To derive a measure analogous to admixture for morphology, we subtracted the mean normalized morphology index (see below) of each *A. rupestre* populations from the maximum value among *A. rupestre* populations, and subtracted the lowest *A. barrelieri* population value from the mean for each *A. barrelieri* population. These values were regressed onto distance to the nearest heterospecific population.

### 2.3 RADseq and linkage map estimation

To create a recombination map for *A. barrelieri* × *A. rupestre* hybrids, we genotyped the F_2_ population using RADseq, carried out as described previously (Duran‐Castillo et al., 2021). DNA from each F_2_ plant, both F_1_s and the parents was digested with *Sbf* I and ligated to indexed P1 adapters (Etter et al., 2011). DNA from ≤96 individuals with different P1 indices was pooled to create a sub‐library, sheared and size fractionated to 250–550 bp. Indexed P2 adapters were ligated to repaired sheared ends so that each sub‐library carried two P2 indices and each P1‐P2 index combination was unique to an individual. After 16 rounds of PCR amplification, sub‐libraries were pooled and sequenced together (100 bp paired‐end Ilumina) at Edinburgh Genomics.

We assigned Illumina reads to individuals according to their index sequences, trimmed adapter sequences and removed low quality sequences with Stacks Process_radtags (Catchen et al., 2013) before mapping reads to the *A. majus* reference with GSNAP (Wu et al., 2016). SNPs were then called with reference to the F_2_ as a whole using the remainder of the Stacks pipeline. All parameters values used in Stacks and GSNAP were as detailed in Duran‐Castillo et al. (2021). Fragments from the same side of a specific *Sbf* I site all begin with the same sequence (the remainder of the *Sbf* I site) but end at different distances from it, reflecting shearing of *Sbf* I‐digested DNA during library preparation. Coverage of paired‐end reads on the RADseq locus was therefore lower distal to each *Sbf* I site than adjacent to it. To maximize information contained in the reads from a locus, while minimizing the possibility of wrongly calling a heterozygous SNP because of insufficient coverage, we used custom scripts to bin all SNPs within 1.5 kb of each other (i.e. at the same RADseq locus) and to infer which combinations of SNPs represented each haplotype (allele) at that locus from co‐segregation of SNPs within the bin in the F_2_ population (we assumed the probability of recombination within a 1.5 kb locus was negligible). Because the parents are outbreeding and potentially heterozygous, the F_2_, which was produced by crossing two self‐incompatible F_1_ plants, could segregate up to four alleles. We therefore inferred the parental origin of each segregating allele, where possible, by comparison to the F_1_ and parental genotypes. We were then able to assign a genotype call for the locus to each F_2_ individual.

A draft linkage map was made using Joinmap v5 (van Ooijen, 2011), with all distinguishable alleles of a locus coded individually. Loci with alleles assigned to the wrong parent were identified with R/qtl (Broman et al., 2003) and corrected, while 14 F_2_s with sequence coverage that proved insufficient for reliable calling of heterozygotes (i.e. that suggested recombination events to both sides of multiple loci) were removed. A final linkage map was estimated from the revised data by assigning loci to linkage groups at LOD ≥6.0, and positioning them with two rounds of regression mapping at LOD ≥10.0 with Kosambi correction for distances. Recombination and physical maps were visualized with the karyoploteR package (Gel & Serra, 2017).

### 2.4 QTL analysis

Though the mapping parents were heterozygous at many marker loci with more than two segregating alleles, we assumed that that they were more likely to be homozygous for mutations underlying consistent species differences in morphology. To increase the power of QTL detection, we therefore reduced the number of F_2_ genotypes to be compared by recoding genotypes to reflect only the species origin of alleles. QTL analysis was performed in R/qtl (Broman et al., 2003). Positions of likely QTL were identified by multiple imputation with the stepwiseqtl function at 1 cM intervals and a conservative genotype error rate estimate of 0.1%. stepwiseqtl was allowed to test models with interaction between QTL and to add interacting QTL pairs but returned additive‐only models as the best fit to all traits. It estimated QTL significance from 1000 genotype–phenotype permutations. The effect of each QTL was estimated with the others for the same trait fixed using the fitqtl function and 0.95 and 0.99 confidence intervals for positions were taken as the points at which the LOD score fell from its maximum by 1 or 2, respectively.

### 2.5 Other statistical analyses and ordination

To compare the morphologies of plants in different populations (wild or glasshouse‐grown and species or experimental hybrids), we derived a 1D morphology index. Character values were first normalized by subtracting the mean value for the character and dividing by the standard deviation, to give them equal initial weights. Then least genetically admixed representatives of *A. barrelieri* and *A. rupestre* identified by Structure analysis (36 and 16 plants, respectively, measured in the field), were used as a training set. Their character values were subjected to linear discriminant analysis in PAST, which adjusted the weighting of each character by a coefficient (discriminant factor) so that two species were maximally separated along a single axis. Weightings are given in Table 1. We applied these weightings to the normalized character values for individuals from other populations (greenhouse‐grown *A. barrelieri*, *A. rupestre* or experimental hybrids) to calculate their morphology index values. We also used PAST to apply principal component analysis (PCA) to normalized character values for all *A. rupestre* and *A. barrelieri* plants measured in the field. As expected, the weightings from discriminant analysis were correlated with the contribution of each character to the first principal component PC1 in PCA (*p* = .03, *R* ^2^ = .46). To examine association of the genetics and morphology of *A. barrelieri* and *A. rupestre* with environmental variables at their collection sites, we used canonical correspondence analysis (CCA), because genetics and morphology were correlated with each other. Each plant was placed in one of four genetic categories according to the proportion of its genome assigned to *A. barrelieri* by Structure at *K* = 5. Categories Gen1‐Gen4 represented assignment of <25%, 25%–49%, 50%–74% and ≥75% *A. rupestre* genome, respectively. Each individual was also assigned to one of four morphological categories according to its morphology index value: categories Morph 1–4 represent morphology index values <−1.00, −1.00 to 0, 0–1 and >1, respectively. Habitats at the collection sites were then designated as low cover (≤50% vegetation cover) or High cover (>50% cover), either Low slope (<45°) or High slope (45°–90°), and substrate classified as rock, wall or soil. CCA was carried out in PAST.

**TABLE 1:** Morphological character differences between *A. barrelieri* and *A. rupestre*.

	*A. barrelieri*	*A. rupestre*	*p* ^b^	Heritability^c^	PC1^d^	Weight^e^
	Mean^a^ (*n* = 105)	Mean^a^ (*n* = 154)				
ln plant height (cm)	4.2	3.3	.000	0.63	0.51	0.84
Internode L (cm)	2.32	1.49	.000	0.36	0.16	0.62
ln branch index	−2.8	−1.5	.000	0.44	−0.37	−0.09
Leaf L/W ratio	2.0	1.5	.001	0.68	0.22	0.04
ln leaf A (cm^2^)	5.2	2.9	.000	0.70	0.47	0.26
ln no. flowers	1.90	0.48	.000	0.31	0.35	1.35
Flower L (cm)	2.3	2.1	.016	0.72	0.06	0.28
Pedicel L (cm)	0.44	0.54	.219	0.25	Not included	
Sepal L (cm)	0.60	0.77	.000	0.53	−0.23	−0.62
Dorsal petal angle	34°	26°	.023	0.39	0.26	0.46
Dorsal petal L (cm)	0.99	0.67	.000	0.67	0.27	0.17

^a^ Mean values for individuals of the same species measured in the glasshouse (see Table S1 for sampling among populations).

^b^ Student's *t*‐test probabilities for the means being the same.

^c^ Heritability estimated as the proportion of variance accounted for by sharing a mother. It is conservative in not accounting for genetic variation between fathers of offspring from the same mother but is unable to separate any maternal from genetic effects.

^d^ Eigenvectors for the contribution of each trait to PC1 for plants measured in the field (traits were normalized to give equal weightings before PCA) *A. barrelieri n* = 88, *A. rupestre n* = 64.

^e^ Weighting coefficient for the contribution of each character to the 1D morphology index for plants measured in the field.

To test whether the distribution of RAD loci was non‐random, the frequency distribution of the physical distances between adjacent loci was compared to simulated exponential distributions with the same mean, as expected of randomly located loci, using the LK.test function of package EWGoF (Krit et al., 2016). Clustering of polymorphic RAD loci was tested in a similar way, using the number of fixed loci between neighbouring polymorphic loci, and a *χ* ^2^ comparison of observed frequencies with those expected for randomly distributed polymorphisms (an exponential distribution of non‐polymorphic site numbers). To test correspondence of QTL in two mapping populations, the closest markers of known sequence flanking the most likely position of each *A. majus* × *A. molle*, QTL from a previous mapping experiment, was used to identify an interval in the *A. majus* reference likely to contain the QTL. For the five leaf‐shape (LfLW) QTL, the intervals together covered 60 Mb (11.5% of the genome). Three of the five LfLW QTL from this study fell within these intervals. This is unlikely to have occurred by chance (*p* = .01, taking the binomial probability of three successes from five trials at the individual success rate of 0.115 expected for QTL that were randomly distributed in the second population). Similarly, the single trichome‐distribution QTL in this study fell within the 10 Mb interval identified previously for the QTL underlying the same trait in an *A. molle* × *A. majus* population (*p* = .02).

## 3 RESULTS

### 3.1 Morphologically diverse *Antirrhinum* species are closely related

Previous sampling of the *Antirrhinum* taxa in southeast Spain might not have been sufficiently deep nor unbiased to reveal the extent to which they are genetically delimited because phylogenetic analysis using multi‐locus nuclear markers had failed to support most morphological taxa, though it consistently nested them together within the lowland clade (taxonomic section *Antirrhinum*; Figure 1a; Duran‐Castillo et al., 2021; Otero et al., 2021; Wilson & Hudson, 2011). To examine the relationships of the taxa further, we focussed on the lowland ecomorph *A. barrelieri* and the alpine *A. rupestre*, which occur in sympatry and are endemic to the Alpujarra region of southeast Spain (Figure 1). We sampled across the whole region and did not exclude samples on the grounds of their morphology (e.g. if they showed intermediate morphologies suggestive of hybrids; Table S1, Figure S1). To place the diversity of these two species in a wider context, we also sampled populations of the three closest neighbouring species: the alpines *A. hispanicum* and *A. mollissimum*, which replace *A. rupestre* to the west and east, respectively, and the lowland *A. tortuosum*, which has a wide range in southeast Spain outside the Alpujarra (Rothmaler, 1956; Sutton, 1988; Webb, 2010).

At two diagnostic plastid loci, all individuals of the five species were found to carry the same derived haplotype that is characteristic of the ancestral alpine lineage (see Section 2). This suggests that all the species share a recent common maternal ancestor, possibly though hybridisation of ancestral alpine and lowland lineages.

**FIGURE 2:**
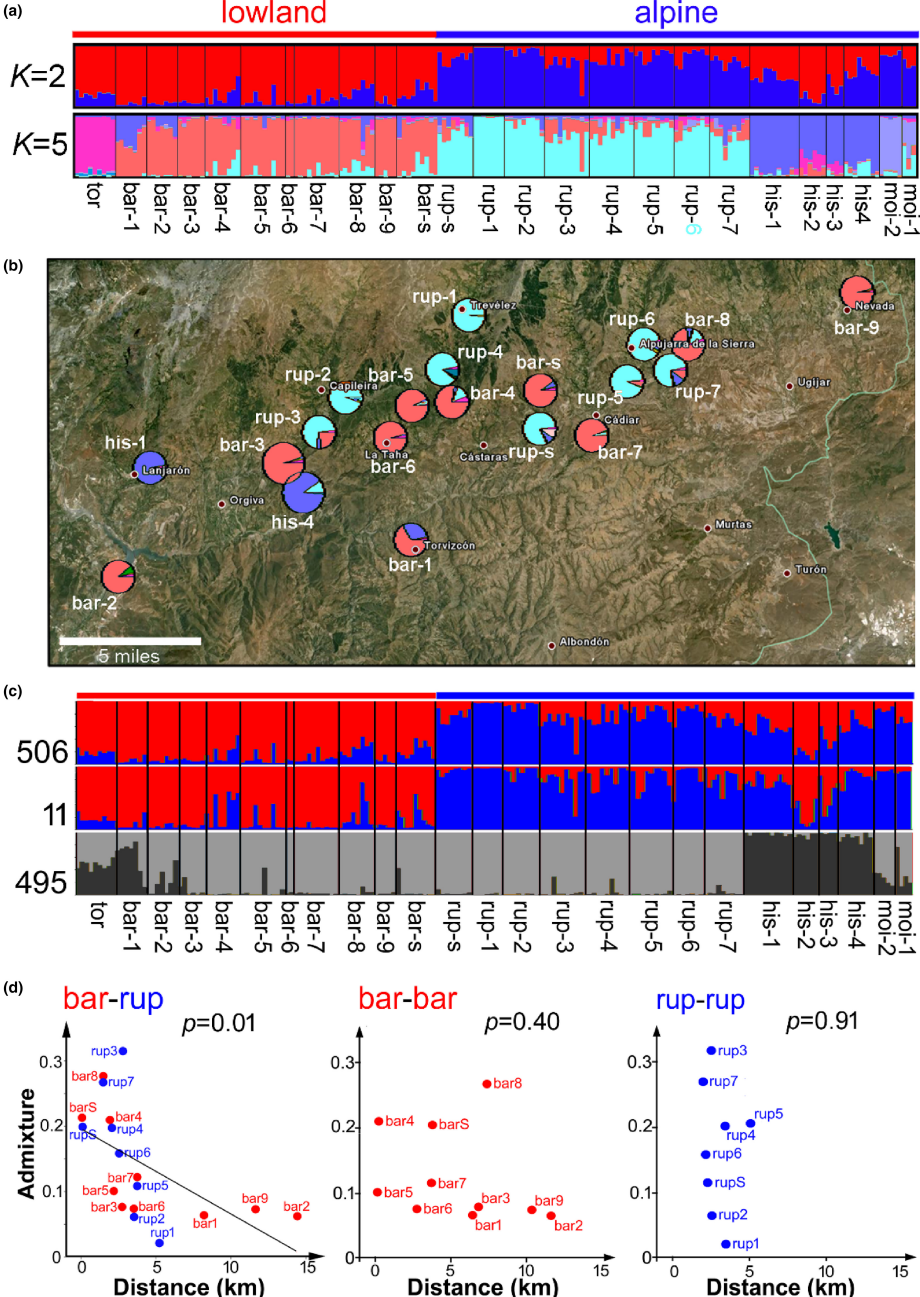
Genetic differentiation of local *Antirrhinum* species. (a) The proportions of each individual's genome that was assigned to each of two (*K* = 2) or five (*K* = 5) ancestral populations are shown. Individuals are grouped according to location and the contribution of each ancestral haplotype is shown in a different colour along the *y*‐axis. (b) Locations of sampled *A. barrelieri* and *A. rupestre* populations are indicated with pie‐charts showing the mean genetic composition of each population, coloured as for *K* = 5 in (a). (c) Assignment at *K* = 2, as in (a), using all 506 loci, only the 11 loci with most biased distribution of alleles between *A. barrelieri* and *A. rupestre*, or the remaining 495 unbiased loci. (d) The average proportion of admixture in *A. barrelieri* and *A. rupestre* populations at *K* = 2, as in (a), plotted against minimum distance to a population of the other species. The *p*‐value is for no linear relationship. In contrast, admixture is not related to distance to the nearest population of the same species, as shown in the two panels to the right.

To examine relationships between taxa, populations of each were genotyped at 506 nuclear AFLP loci and the genomes of individuals assigned to inferred ancestral populations using Structure software. We first examined assignment to one of two populations (*K* = 2 in Figure 2a) to test whether genetic clusters corresponded to alpine and lowland ecomorphs. In this case, all accessions of *A. tortuosum* and *A. barrelieri* (lowland) were assigned to one cluster and all alpines (*A. mollissimum*, *A. rupestre* and *A. hispanicum*) to the other, with the exceptions considered below. Genetic variation therefore broadly corresponds to ecomorph type. However, genetic admixture was suggested for many individuals and was most marked in three *A. hispanicum* populations (his‐1, his‐2 and his‐4 in Figure 2b). The his‐1 and his‐4 populations share about half of their genomes with the lowland cluster, possibly through hybridisation with local *A. barrelieri*, while the his‐2 population, which is located on the northern flank of a separate, more western mountain range, has more than half its genome assigned lowland ancestry, possibly through hybridisation with neighbouring *A. tortuosum*. Because our sampling strategy was focussed on *A. barrelieri* and *A. rupestre*, we did not test possible causes of admixture in *A. hispanicum* further. The model best fitting the genetic data was obtained with five populations (*K* = 5, Figure 2a), when members of each recognized species were assigned mostly to separate clusters. Though this provided genetic corroboration for the species‐level taxonomy, most individuals of *A. barrelieri* and *A. rupestre* showed some admixture with the other species. To assess the proportion of loci responsible for assignment of these two species to separate genetic clusters at *K* = 2, the loci showing the most significant bias between the two species (F_*ST*_ outliers, see Section 2) were removed sequentially. The two species failed to be assigned to different clusters after removal of only the 11 most biased loci (2% of the total). These loci were not only necessary but also sufficient to distinguish the two species from each other (Figure 2c).

The genetic admixture inferred for many individuals of *A. rupestre* and *A. barrelieri* could be explained by sharing of ancestral polymorphism through lineage sorting, in which case we do not expect it to be related to physical separation of the two species. Alternatively, admixture could reflect gene flow; in which case it may be higher where populations of the two species are closer together. Indeed, we found a significant (*p* = .013) relationship between the level of admixture assigned to populations at *K* = 2 and the proximity of the other species (Figure 2d), consistent with gene flow between them.

### 3.2 Closely related species are defined by a suite of genetically determined characters

**FIGURE 3:**
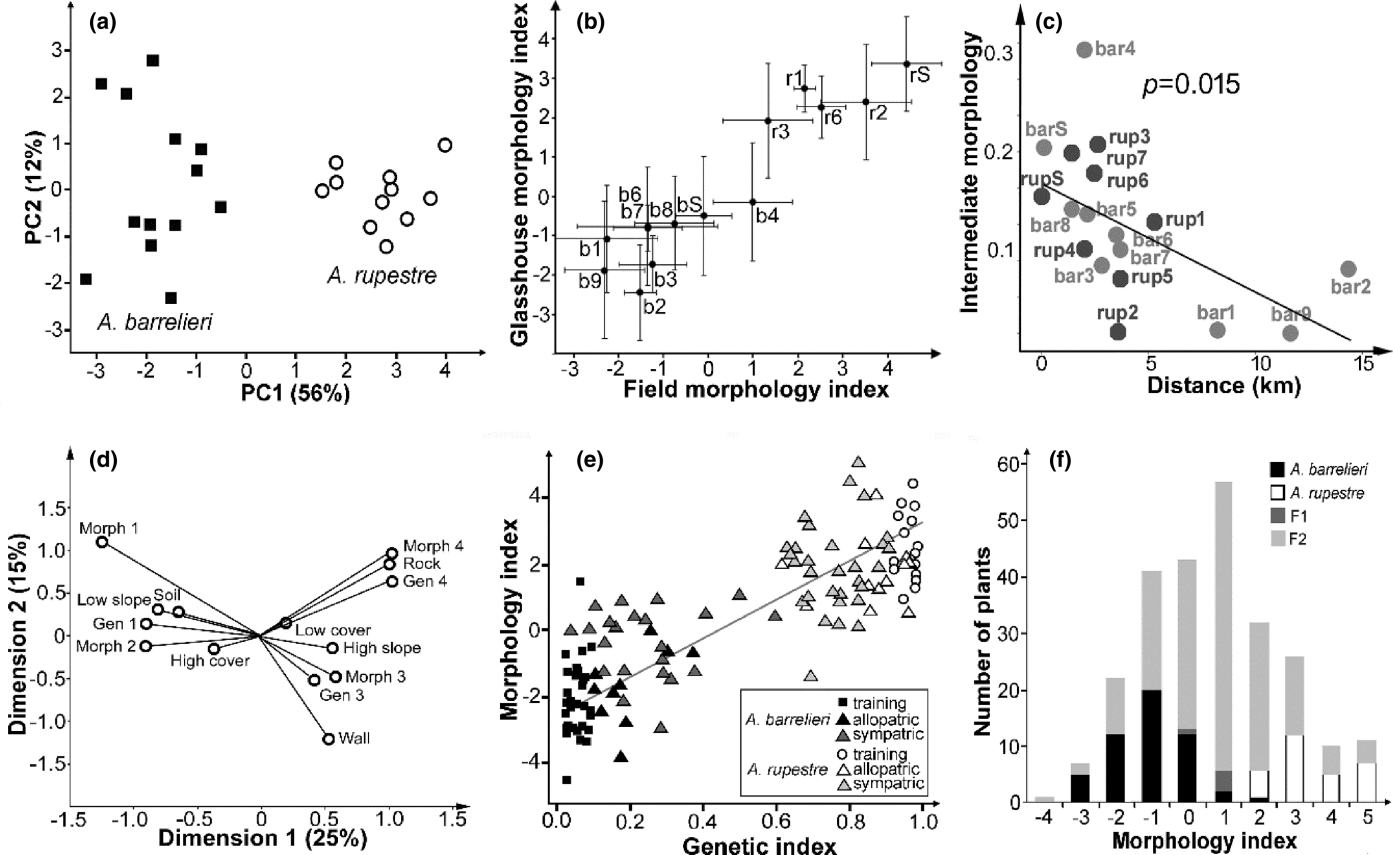
Morphological variation within *A. rupestre* and *A. barrelieri* correlates with genetics and habitat. (a) Principal components analysis of morphological variation in *A. barrelieri* and *A. rupestre* populations (each point represents the mean for one population). (b) Correlation between morphology of populations in the field and their progeny in a glasshouse (mean population morphology indices ±1 SD; *b A. barrelieri*, *r A. rupestre*). (c) The relationship of intermediate morphology to proximity of the other species. Intermediate morphology is the difference between the morphology index of each population and the most extreme value for the species. Distance is the distance of each population to the nearest population of the other species. (d) Association of morphology and genetics with different features of habitat represented as the first two dimensions of a canonical correspondence analysis (CCA). Categories used for each variable are detailed in Section 2. All associations are significant with *p* ≤ .04. (e) Morphology index plotted against genetic index (the proportion of the genome assigned to *A. rupestre* in Structure analysis at *K* = 2). ‘Training’ plants were the least genetically admixed individuals used to derive the morphology index. *p* = 2 × 10^−34^ and *R* = .84 for all individuals, and *p* = 6.0 × 10^−12^ and *R* = .69 with training plants excluded (the regression line is the same for both). (f) Morphological variation in F_1_ and F_2_ hybrids of *A. rupestre* × *A. barrelieri* compared to allopatric representatives of the parent species. Samples sizes are given in Section 2 and in Table S1.

We further examined the morphological differentiation of alpine and lowland ecomorphs in the region using *A. barrelieri* and *A. rupestre*. We first measured members of each population in the field (a total of 88 *A. barrelieri* and 64 *A. hispanicum*) for characters that are known to be heritable and contribute most to the distinction between alpine and lowland ecomorphs in the genus *Antirrhinum* as a whole (characters are given in Table 1; Wilson & Hudson, 2011). Variation in each character was normalized to a mean of 0 and standard deviation of 1 and the major axes of character covariation were then identified as principal components (PCs). The two species formed separate clusters along PC1, which explained most (56%) of the variance between individuals (Figure 3a). All characters, except flower length made a substantial contribution to PC1, with the largest coming from plant height, leaf area, degree of shoot branching and the number of flowers in the inflorescence (eigenvectors are given in Table 1). All except branching and sepal length were positively correlated, indicating that *A. barrelieri* is taller, has larger leaves with narrower shapes, smaller sepals and more flowers per inflorescence than *A. rupestre*, and also has proportionately shorter lateral branches. In contrast to PC1, PC2 described mainly variation within species. These findings suggest that the divergence of the two species involved multiple morphological characters and paralleled the earlier divergence between ancestral alpine and lowland ecomorphs.

To examine the heritability of characters measured in wild plants, we grew their progeny together in a glasshouse from field‐collected seeds (a total of 154 plants from *A. rupestre* seed parents and 105 from *A. barrelieri*). Under greenhouse conditions, the species were found to differ significantly for all characters except pedicel length (Table 1), further supporting the view that the classical species taxonomy is based on a suite of genetically determined characters involving different parts of the plant. A further consistent difference was that *A. rupestre* produced trichomes from all leaves and internodes, in common with all other alpine *Antirrhinum* species, whereas leaf blades and internodes of *A. barrelieri*, like those of most lowland species, lacked trichomes beyond the seedling stage (Tan et al., 2020). However, one notable difference from most other alpines was that *A. rupestre* did not have white petals; instead they were magenta, like those of *A. barrelieri* and most other lowland species (Figure 1c,g; Whibley et al., 2006).

To derive a morphology index with which to compare different sets of plants, each character was weighted so that the least genetically admixed members of the two species were maximally separated along the primary axis of character co‐variation (Table 1). Applying this index to populations showed that the morphologies of populations in the field were highly correlated with those of their glasshouse‐grown offspring (*R* ^2^ = .88, *p* < 1 × 10^−4^, Figure 3b), suggesting that morphological variation was determined largely by genetic differences, rather than differences between the environments in which the plants had grown.

Genetic admixture between *A. barrelieri* and *A. rupestre* had proved to be correlated with physical proximity to the other species. Similarly, we found that populations with more intermediate phenotypes tended to occur closer to populations of the other species (Figure 3c), which is also suggestive of gene flow. Even so, intermediate morphologies were uncommon in the wild (Figure 3f).

### 3.3 Morphology is associated with habitat

Because populations of *A. rupestre* and *A. barrelieri* are interspersed in a small geographic area, we tested whether each species is associated with different features of the local landscape. Three environmental factors—substrate type, slope and vegetation cover—were found to be associated with each other and with plant morphology (Figure 3d, *p* = .02 for no overall association and ≤.04 for each environmental factor separately). This confirmed that *A. rupestre* tended to grow in rock faces that have high slopes and low vegetation cover and *A. barrelieri* in more level soils in greater competition with other species. Though relatively rare, plants with intermediate genotypes and morphologies were usually found in intermediate environments, including stone‐built walls. Three other environmental factors—elevation, soil pH and soil moisture content—showed no significant correlation with either plant morphology or genetics. Niche differentiation can therefore be attributed to physical substrates, which have a mosaic distribution in the Alpujarra region (Ruiz Montez et al., 2011).

### 3.4 Multiple genes underlie morphological divergence of closely related species

To understand better the genetic basis for the morphological divergence of *A. barrelieri* and *A. rupestre*, we crossed a representative of each species and grew their progeny together under the same conditions. F_1_ and most F_2_ progeny were intermediate in overall morphology (Figure 3f) and for most characters considered separately (Figure S2), suggesting that multiple genes underlie variation in each character. A few characters showed relatively little transgressive segregation in the F_2_ (notably leaf shape, leaf size and sepal length), implying that most of the alleles in one of the species increase the character. Notably, intermediate phenotypes were the most common in hybrids, contrasting with the variation seen in wild populations, where intermediate morphologies are rare (compare Figure 3c,f). Unlike the continuous characters, trichome distribution was inherited as if determined by a single locus at which the *A. barrelieri* allele is a dominant trichome suppressor: leaf blades and stems were glabrous in F_1_ hybrids and in around three‐quarters of F_2_ hybrids (123 of 169 plants, *χ* ^2^ *p* = .51 for a 3:1 segregation model).

The co‐variation in characters between *A. rupestre* and *A. barrelieri* could reflect developmental constraints, for example, the effects of pleiotropic or closely linked genes, in which case, character correlations should be maintained in F_2_ hybrids. However, only four significant relationships survived in the F_2_. These involved direct correlations between plant height and leaf narrowness, branching and flower number, and the lengths of sepals and pedicels, and an inverse correlation between branching and flower number (Figure S2). Even in these cases, correlations were lower in the F_2_ than in the species grown under the same conditions. Because parental character combinations are lost or reduced in the F_2_, the differences of *A. rupestre* from *A. barrelieri* appear to be determined largely by loci that are able to segregate independently—that is by extended haplotypes that can be broken down by recombination.

### 3.5 Recombination in *A. barrelieri* × *A. rupestre* hybrids

**FIGURE 4:**
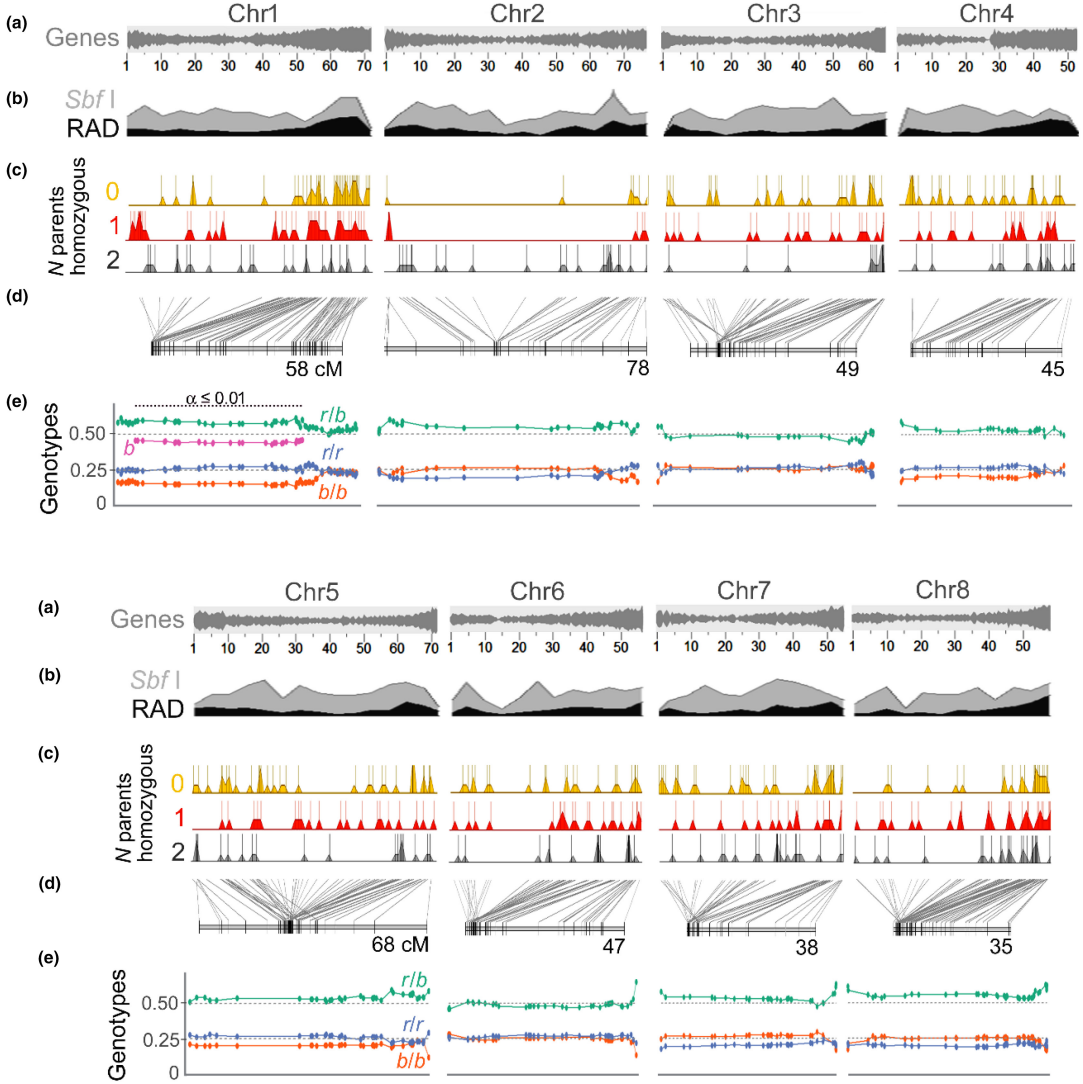
Polymorphism and linkage in *A. barrelieri* and *A. rupestre* genomes. (a) Gene density plotted along a physical map of the *A. majus* genome. Coordinates are in Mbp. (b) Sliding‐window plots of the predicted density of *Sbf* I sites (light grey) and RADseq loci recovered from both *A. barrelieri* and *A. rupestre* (black)—sequences unique to one species were not included. (c) Physical locations of polymorphic RADseq loci that were homozygous in both parents (2), one parent (1) or neither (0). (d) A linkage map of RADseq loci. Slanted lines compare the map position of each marker with its physical position in the *A. majus* genome. (e) Proportions of genotypes in the F_2_ population compared to the expected proportions of 0.5 for heterozygotes and 0.25 for each homozygote in the absence of distorted transmission (*r* = *A. rupestre*, *b* = *A. barrelieri*). The proportion of alleles from *A. barrelieri* is plotted separately for the region of Chr1 showing distorted genotype ratios.

To further examine the genomic basis for divergence of *A. barrelieri* from *A. rupestre*, we genotyped both species and their F_1_ and F_2_ hybrids by RAD‐seq around *Sbf* I restriction sites. One thousand loci were recovered from both species, with a mean length of 632 ± 11 bp (SE). We first examined the extent to which the distribution of RAD loci represented the genome as a whole. They were not found randomly distributed across the genome, being more frequent in gene‐rich regions (Figure 4a,b; Figure S3A,B). This bias is consistent with the GC content of *Sbf* I sites (75%) being closer to that of *Antirrhinum* coding sequences (44%) than to the non‐coding component of the genome (34%; Li et al., 2019). Of the RAD loci, 568 (57%) were found to be polymorphic within or between the parents (Figure 4c). We also examined whether polymorphisms between the parents differed across the genome, and found for that the probability of adjacent RAD loci being polymorphic was not significantly different from the expectation for polymorphisms randomly distributed across loci (*χ* ^2^ *p* = .43; Figure S3C). Therefore, although the distribution of RAD markers was biased towards gene‐rich regions, the markers represented all chromosomes at similar densities (Figure 4a–d).

To examine the inheritance of genomic regions in hybrids and to map loci underlying morphological variation between the parent species, we estimated a linkage map from RADseq loci of the F_2_ population (*n* = 216). Of the 508 polymorphic loci with <25% missing data across the mapping population, 462 (81%) could be assigned map positions with an odds‐ratio ≥10^5^. The ability to map a locus was not affected significantly by distorted transmission (see below) or genome position but increased with the number of segregating alleles (Table S2). This suggests that marker representation in the map is related to information content and not biased towards particular parts of the genome, other than the gene‐rich regions that are over‐represented in RADseq.

The map comprised eight linkage groups, covering an estimated 93%–96% of the genome (Figure 4e; Chakravarti et al., 1991). Its total length of 418 cM was 21% shorter than a map made from hybrids between two *A. majus* cultivars (562 cM; Schwarz‐Sommer et al., 2010), suggesting that sequence differences between *A. rupestre* and *A. barrelieri*, which are assumed to be larger than differences within *A. majus*, have relatively little effect on the overall frequency of recombination. Map positions of markers were mostly co‐linear with their physical locations in the *A. majus* reference sequence (Figure 4d). The very few exceptions can be explained by rearrangements in both mapping parents relative to the reference, assignment of markers to incorrect map positions or reference mis‐assembly. Variation in recombination frequency along chromosomes was reflected in clustering of physically dispersed markers in the linkage map, as observed for hybrids between or within *Antirrhinum* species previously (Feng et al., 2009; Li et al., 2019; Schwarz‐Sommer et al., 2003). However, we found no evidence for large blocks of non‐recombining markers characteristic of chromosome inversion polymorphisms between the parents. For example, the densest cluster, towards the middle of Chr5, contained 50 markers within 5.1 cM, yet only four groups of either two or three markers showed no internal recombination in the mapping population. The map also failed to provide any evidence, in the form of pseudolinkage between markers from different chromosomes (Farré et al., 2011), for chromosome translocation polymorphisms that can also suppress recombination and reduce hybrid fitness.

Many barriers to gene flow, including those involved in genetic incompatibilities or pollen competition, can change the proportions of alleles and genotypes between F_1_ and F_2_ generations causing transmission ratio distortion (TRD; reviewed by Fishman & McIntosh, 2019). In the *A. barrelieri* × *A. rupestre* F_2_, only one region, in Chr1, showed significant TRD (Figure 4e). This involved a moderate (~11%) reduction in the proportion of *A. barrelieri* alleles. The proportions of F_2_ genotypes were not inconsistent with the biased allele frequencies (*χ* ^2^ *p* = .09 at the most distorted locus), suggesting that distortion had occurred in the haploid phase of the F_1_ (e.g. in its gametes). The alternative possibility that TRD reflected a reduced ability to map *A. barrelieri* sequencing reads in RADseq (reference bias), appeared unlikely, given that much of Chromosome 1 but no other chromosome, was affected. Overall TRD involved only around 3% of the linkage map, which was similar in extent to TRD in hybrids between *A. majus* cultivars (4%; Schwarz‐Sommer et al., 2010) and considerably less than in hybrids between lowland *A. majus* and alpine *A. molle* (35%; Schwarz‐Sommer et al., 2003). The low proportion of the genome affected by TRD and its modest magnitude suggest that its causes would have little effect on gene flow between *A. barrelieri* and *A. rupestre* populations.

### 3.6 Mapping loci underlying morphological divergence

**FIGURE 5:**
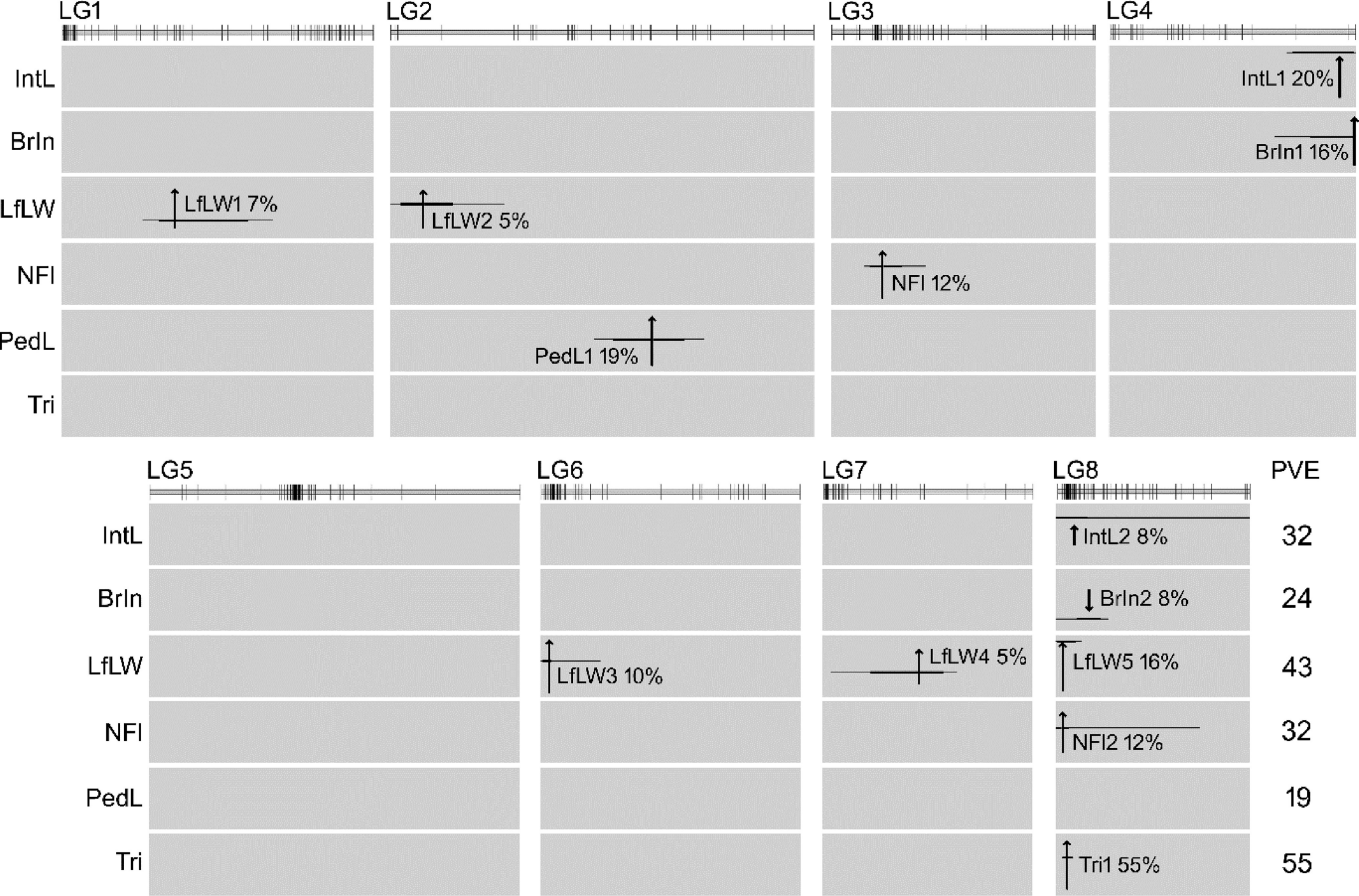
QTL underlying morphological differences between *A. barrelieri* and *A. rupestre*. The most likely position of each significant QTL is shown by an arrow with a length proportional to the magnitude of the QTL effect and an orientation reflecting the direction of parental alleles (upwards pointing shows that the *A. barrelieri* allele increase the character value). Estimates of 0.95 and 0.99 confidence intervals for QTL positions are shown by thick and thin horizontal lines, respectively. The position at which the line cuts the arrow represents the relative trait value estimated for heterozygotes (e.g. the *A. barrelieri* allele at locus LfLW5 is fully dominant). The percentage of F_2_ variance explained (PVE) is given for loci individually and in total. No significant QTL was detected for the remaining traits. The positions of markers used in QTL analysis are shown on the linkage map above the positions of the QTL.

The inheritance of morphology in hybrids, with the exception of trichome distribution, had suggested that each character involved multiple genes that were able to segregate independently. To test this further, we used the F_2_ population to scan for genes underlying parental differences as quantitative trait loci (QTL). We detected significant QTL for six characters (Figure 5) and the number of QTL detected for each was consistent with predictions: multiple loci (between two and five) for each character except trichome distribution (one QTL). With one exception, all loci had only alleles that contributed to the differences between parents. The exception involved branching, for which the two significant QTL had opposite effects (Figure 5). This can help explain why branching showed more extreme transgressive segregation in the F_2_ than most other characters (Figure S2). QTL were spread over seven of the eight *Antirrhinum* chromosomes, and half of them did not overlap in position with a QTL for another character, consistent with loss of parental character correlations by segregation in F_2_ hybrids. However, QTL for branching and internode length mapped to the same part of Chr2 while QTL for five different characters were found in the same region of Chr8. Of the characters with co‐located QTL, only two showed a significant correlation in the F_2_ population: branching was inversely related to the number of flowers, which may reflect the same region of Chr8 containing loci (BrIn2 and NFl2) that act in opposite directions (Figure 5). However, the QTL that we detected explained less than half of the variation in each quantitative character (between 19% and 45% of the F_2_ variance; Figure 5). Therefore, the lack of significant correlation between other characters with co‐located QTL, and the absence of co‐located QTL for characters that remained correlated in the F_2_, may reflect the action of minor‐effect QTL or epistatic interactions that we were unable to detect with a mapping population of this size.

To test whether the QTL underlying variation between *A. rupestre* and *A. barrelieri* could correspond to genes involved in the earlier alpine‐lowland divergence, we compared positions of QTL in this study with those detected previously in hybrids between *A. majus* (lowland ecomorph) and *A. molle* (an early‐diverged alpine from northern Spain; Schwarz‐Sommer et al., 2003; Yang, 2006). Only three characters had been analysed in a similar way in both populations (leaf shape, pedicel length and trichome distribution). Four leaf shape QTL had been detected in *A. molle* × *A. majus* hybrids compared to five for *A. rupestre* × *A. barrelieri* (Table 2). However, three of these QTL located to the same genome intervals in the two populations—a correspondence that was unlikely to have occurred by chance (*p* = .01; see Section 2 for binomial probabilities), suggesting that the divergence of *A. rupestre* and *A. barrelieri* might have involved some of the same genes as the earlier lowland‐alpine split. Alleles at all three loci also acted in the same direction in the two comparisons, with the alpine (*A. rupestre* or *A. molle*) allele making leaves broader in shape (i.e. decreasing length: width ratio), as expected for reused mutations. Similarly, the only QTL for trichome distribution in each population was found at the same location in Chr8 (*p* = .02) and the alpine allele increased leaf trichomes in both cases. This location includes the *Hairy* trichome‐suppressor gene, mutant alleles of which appear to have been reused in the parallel divergences of alpine from lowland ecomorphs (Tan et al., 2020). In contrast, neither of the two pedicel length QTL detected in this study mapped to the single QTL identified previously for this character.

**TABLE 2:** Correspondence of QTL in parallel divergences.

Trait	*A. molle* × *A. majus*						*A. rupestre* × *A. barrelieri*				
	QTL	Chr	Start^a^	End^a^	Dir^b^	PVE^c^	QTL	Chr	Position	Dir^b^	PVE^c^
Leaf shape	LePC2.1^d^	1	29.9	60.8	↑	10	LfLW1	1	55.9	↑	7
	LePC2.4	1	1.1	10	↑	7	LfLW5	1	7.6	↑	16
	LePC2.3	2	65	75	↑	11	LfLW2	2	0.6	↑	5
	LePC2.2	7	45	55	↑	5	LfLW4	7	49.4	↑	5
							LfLW3	5	1.6	↑	10
Pedicel L	F_1.1_	6	26.5	39.8	↓	15	PedL1	2	68.4	↓	19
	F_1.2_	8	50.9	60.9	↓	21					
Trichomes	DL_totA1	8	40.7	50.7	↓	74	Tri1	8	43.6	↓	55

^a^ Locations of markers (in Mb) flanking the most likely positions of QTL within the *A. majus* reference genome.

^b^ Direction of the QTL alleles: an arrow pointing upwards shows that the lowland allele (*A. majus* or *A. barrelieri*) increases the trait value.

^c^ Percentages of variance for the traits in the F_2_ mapping populations that are explained by the QTL.

^d^ Leaf shape traits in the *A. molle* × *A. majus* F_2_ were described by principal components; PC2 described variation in leaf shape equivalent to length:width ratio.

## 4 DISCUSSION

### 4.1 Relationships between *Antirrhinum* species in southeast Spain

The traditionally recognized *Antirrhinum* species from the Alpujarra region can be delimited genetically. However, only around 2% of nuclear markers distinguish *A. rupestre* from *A. barrelieri*. Though this genetic similarity is consistent with recent divergence from a common ancestor, gene flow may contribute to it because genetic admixture and intermediate morphologies tend to be higher where the species grow closer together.

Despite being genetically very similar, *A. rupestre* and *A. barrelieri* remain distinct for a suite of heritable morphological characters that typify alpine and lowland ecomorphs in the genus as a whole. These character combinations further correlate with habitat, particularly substrate, as expected of ecological adaptations, and mirror alpine adaptation in other genera (Billings, 1974; Körner, 2003).

While species in the ancestral group of alpines grow at the highest elevations for *Antirrhinum* (>2000 m) in rock‐faces and screes, those in southeast Spain extend to lower levels (Rothmaler, 1956; Sutton, 1988; Webb, 2010). Here plants may face similar pressures related to growth on exposed rock‐faces as in alpine or sub‐alpine environments—in fact steepness appears a better predictor than elevation of alpine ecosystems generally (Körner et al., 2011). Other characters that are not shared with ancestral alpines (e.g. pink flowers in *A. rupestre*) may involve adaptation to differences associated with elevation.

The role of mountains as cradles of speciation is well documented and attributed to ecological opportunity coupled with geographic isolation (reviewed by Hughes & Atchison, 2015). Different mountains present similar opportunities (involving, for example, temperature, seasonality, irradiation and substrate) and the potential for repeated eco‐geographic isolation and therefore for parallel divergence of ecotypes (e.g. Bertel et al., 2018; Bohutínská et al., 2021; Konečná et al., 2021) or species—for example of African *Dendrosenecio* (Tusiime et al., 2020).

### 4.2 Morphological divergence involved multiple unlinked genes

With the exception of trichome distribution, each of the characters that distinguish *A. barrelieri* from *A. rupestre* involves multiple, unlinked genes so that parental character combinations are readily broken down in hybrids. We detected at least eight loci involved in species divergence as 13 QTL. However, many more loci are likely to be involved, including undetected minor‐effect QTL, genes underlying less visible characters (e.g. physiological adaptation to substrate) and genes that are polymorphic within species but not between the mapping parents. Indeed, studies of alpine adaptation from lowland ancestors in other angiosperm taxa implicate divergence in form, life history and multiple physiological processes (e.g. Bohutínská et al., 2021; Coughlan et al., 2021; Knotek et al., 2020).

### 4.3 Barriers to recombination

The involvement of multiple genes that can recombine in hybrids raises the question of how parental character combinations could be maintained under conditions of gene flow. We find association of species with substrate, consistent with ecological adaptation, and evidence for ecological selection against hybrids in the low frequency of genetically admixed and morphologically intermediate plants in the wild. In the case of *A. rupestre* and *A. barrelieri*, low hybrid fitness is supported by the large excess of morphologically intermediates in experimental F_1_ and F_2_ hybrids over the wild populations (Figure 3f). However, ecological selection against hybrids is unlikely to be the only mechanism maintaining the species because parents divergent for multiple adaptive genes would be unfit if they mated randomly to produce a high proportion of maladapated hybrid offspring (Kirkpatrick & Ravigne, 2002).

Considering potential intrinsic, post‐pollination barriers, we find no evidence in hybrids for chromosome rearrangements or for suppressed recombination across the genome as a whole. Though five QTL that affect different characters are co‐located in two regions (reflecting linkage of multiple genes or pleiotropy), the regions appear to contribute relatively little to maintaining character combinations because correlations are largely lost in segregating hybrids. We further found no evidence for incompatible interactions between parental alleles that reduce hybrid fitness or transmission of recombinant haplotypes: F_1_ hybrids were fertile, F_2_ hybrids viable, and distorted transmission was both moderate in its effect and confined to a region carrying only one of the 13 identified QTL.

Though evidence for postzygotic barriers other than ecological selection were not detected, glasshouse grown offspring of wild *A. barrelieri* or *A. rupestre* (which are obligate out‐crossers) all resembled their mothers rather than intermediate F_1_ hybrids, even in populations close to the other species (see Figure 3b), implying that their fathers were conspecific. How this may occur is unclear; the two species overlap in flowering period and share pollinators (almost exclusively bees; Vargas et al., 2017). One possibility is that the spacing of preferred substrates relative to the limited foraging range of bees, which may be territorial, leads to mainly conspecific pollination.

### 4.4 Reuse of ancestral polymorphisms

We find evidence that four of the genomic regions underlying divergence of *A. barrelieri* and *A. rupestre* might have also involved in an earlier split of ancestral alpine and lowland lineages (represented by *A. majus* and *A. molle*; Schwarz‐Sommer et al., 2003; Yang, 2006), which could reflect involvement of the same genes. Alleles at the four loci also act in the same direction in the parallel evolved alpines, making leaves rounder in shape and hairy, which could be explained either by parallel selection of independent mutations with similar effects or reuse of the same mutations. The hairy leaf character of alpines in the Alpujarra probably involved both allele re‐use and mutation de novo at the *Hairy* (*H*) locus, which is located within the Tr1 QTL. *A. rupestre* and some *A. hispanicum* individuals share the same loss‐of‐function *h* alleles as the earlier‐evolved alpines, while other *A. hispanicum* carry an independent *h* mutant allele that was likely derived recently from the lowland lineage (Tan et al., 2020). Allele re‐use is consistent with introgression from the ancestral alpines, or an origin in incomplete lineage sorting. Population comparisons with whole‐genome data may help distinguish the contribution of both processes, for the *H* gene and for other loci underlying the parallel divergences of ecomorphs (e.g. Lee & Coop, 2017; Martin & Jiggins, 2017).

Introgression of ancestral alpine adaptations in southeast Spain could have been facilitated by lack of strong genetic barriers, other than habitat adaptation, as suggested for *A. rupestre* and *A. barrelieri*. However, much stronger barriers have been detected between earlier diverged alpine and lowland *Antirrhinum* species, as chromosome inversions (Zhang et al., 2005) and extensive TRD (Schwarz‐Sommer et al., 2003), suggesting that existing barriers might have been lost locally. One possibility is that they were purged when an ancestral alpine was swamped genetically by a lowland species. In this scenario, the alpine parent is now represented only by its captured plastid (Jiménez et al., 2005; Liberal et al., 2014; Wilson & Hudson, 2011) and at least part of its alpine‐adapted nuclear haplotype, which was able to survive in parts of the Alpujarra because suitable alpine habitat already existed there (Lonergan & White, 1997; Otero et al., 2021). Genomic analysis of *A. rupestre* and *A. barrelieri* populations should allow this idea to be tested further. Although evidence from other species suggests that reuse of genes in parallel alpine‐lowland divergences is not widespread (e.g. Bohutínská et al., 2021; Konečná et al., 2021), the scenario of alpine haplotype capture differs from other examples in that the adaptation, which we propose leads to ecological isolation, was already assembled, rather than needing to be put together de novo from standing variation in a lowland ancestor.

## AUTHOR CONTRIBUTIONS

All authors designed research. Yvette Wilson collected populations and did plastid and AFLP genotyping, Jo Critchley and Andrew Hudson made RADseq libraries, Matthew Barnbrook called F_2_ genotypes and estimated linkage maps, Mario Durán‐Castillo did QTL and TRD analyses, and Yvette Wilson population and morphometric analyses. Andrew Hudson wrote the paper with input from all other authors.

## CONFLICT OF INTEREST STATEMENT

The authors have no conflict of interest to declare.

### OPEN RESEARCH BADGES

This article has earned an Open Data badge for making publicly available the digitally‐shareable data necessary to reproduce the reported results. The data is available at BioProject ID PRJNA831818 and https://doi.org/10.7488/ds/3465.

## BENEFIT‐SHARING STATEMENT

Benefits from this research accrue from this sharing of these data and results on public databases.

## Supporting information


Appendix S1

## Data Availability

RADseq reads are available from the NCBI Sequence Read Archive (BioProject ID PRJNA831818) and genotype and phenotype data at the University of Edinburgh's public data repository as https://doi.org/10.7488/ds/3456.
