# Supplementary material for: Recent parallel speciation in Antirrhinum involved complex haplotypes and multiple adaptive characters
Source: Mol Ecol. 2023 Aug 21;32(19):5305–22. doi: 10.1111/mec.17101 (PMC10947308; doi:10.1111/mec.17101)
Supplement: Supplementary file 1 — Appendix S1 [file MEC-32-5305-s001.pdf]

MOLECULAR ECOLOGY

Supplemental Information for:

Recent parallel speciation in *Antirrhinum* involved complex haplotypes and multiple adaptive characters

Matthew Barnbrook, Mario Durán-Castillo, Jo Critchley, Yvette Wilson, Alex Twyford, Andrew Hudson

Table of Contents:

**Table S1**	Page 2
**Table S2**	Page 3
**Figure S1**	Page 4
**Figure S2**	Page 5
**Figure S3**	Page 6

MOLECULAR ECOLOGY

Table S1 Population Accessions

Species	Code	Location	Lat (°)	Lon (°)	Elev (m)	Field *N*†	Glasshouse *N*‡
Mixed	barS, rupS	Barranco de la Cimbua	36.96	-3.22	1316	9	4, 4 (9,11)
A. barrelieri	bar-1	Torvizcón	36.88	-3.30	660	7	4
	bar-2	Embalse de Rules	36.87	-3.49	289	9	3
	bar-3	Carataunas to Pampaneira	36.92	-3.38	943	10	5
	bar-4	Barranco de los Llamos	36.95	-3.28	1353	8	4 (7)
	bar-5	Barranco del Tesoro	36.95	-3.28	1346	10	-
	bar-6	Pórtugos	36.94	-3.31	1269	9	6
	bar-7	Cádiar	36.94	-3.18	928	10	4
	bar-8	Yegen	36.98	-3.12	1071	10	5 (5)
	bar-9	Laroles	37.01	-3.01	1060	10	6
A. rupestre	rup-1	Trevélez	37.00	-3.26	1451	8	2
	rup-2	Capiliera to Hoya del Portillo	36.96	-3.34	1792	8	1, 4 (10,4)
	rup-3	Pampaneira	36.94	-3.36	1062	10	5 (10)
	rup-4	4 km south of Trevélez	36.97	-3.28	1462	9	-
	rup-5	near El Golco	36.96	-3.16	1226	9	-
	rup-6	Mecina Bombarón	36.98	-3.15	1154	10	-
	rup-7	Mecina Bombarón to Yegen	36.97	-3.13	1015	10	4 (10)
A. hispanicum	his-1	Lanjarón	36.92	-3.47	635	12	5
	his-2	Alhama de Granada	37.00	-3.99	880	10	5
	his-3	Fornes	36.96	-3.88	861	8	6
	his-4	Tíjola	36.91	-3.37	466	10	3
A. mollissimum	moi-1	Enix	36.88	-2.61	743	10	5
	moi-2	Abrucena	37.13	-2.80	1015	9	6
A. tortuosum	tor-1	Loja	37.16	-4.13	489	10	0

† Field *N* is the number of individuals from each population that was measured in the field. ‡Glasshouse *N* is the number of individuals measured in a glasshouse. For populations distant from the other species, a single offspring was grown in the glasshouse from each wild parent. For other populations, four numbers are given – the first two represent the number of parent plants the second two numbers, in brackets, give the mean numbers of siblings grown from each parent.

MOLECULAR ECOLOGY

Table S2 Comparisons of mapping and non-mapping loci

	Mapped (%)	Unmapped (%)	p[†]
**Missing data**[‡]	14.4±0.67	15.7±1.09	0.20
**Distorted segregation**[§]	28	34	0.19
**Segregating alleles**[¶]			
4 alleles	92	8	*0.0012*
3 alleles	78	2	0.18
2 alleles, neither F1 homozygous	76	24	0.52
2 alleles, one F1 homozygous	62	38	*0.0004*

[†]probabilities, from either Student's *t*-tests or Fisher's exact tests, that the proportions in mapped and unmapped loci are the same. [‡] Mean (±SEM) percentages of missing data (F2 plant x marker combinations without genotype calls). [§] Percentages of loci at which the proportions of possible genotypes differed significantly from the Mendelian expectation, as judged by Fisher's exact test without correction for multiple testing. [¶] Loci classified by the number of segregating alleles and F1 genotypes. Markers that distinguish all four segregating chromosomes ('4 alleles') are under-represented in the unmapped fraction, while those that distinguish only one chromosome from the other three ('2 alleles, one F1 homozygous') are over-represented.

MOLECULAR ECOLOGY

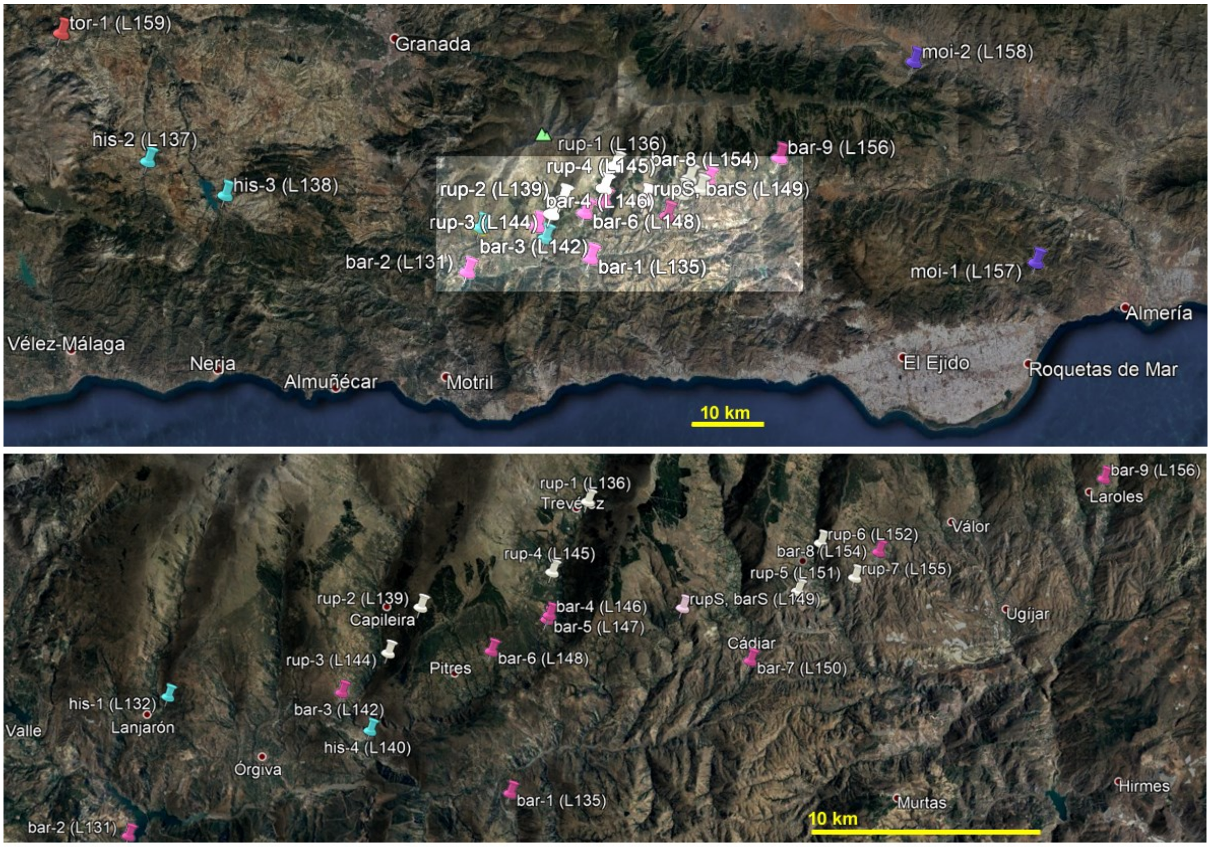

Figure S1 Study sites

The locations of sampled *A. barrelieri* and *A. rupestre* populations are shown by pink and white pins respectively. *A. rupestre* is replaced to the west by *A. hispanicum* (blue pins) and further to the east by *A. mollissimum* (purple). The Alpujarra region (light box in upper map) is show in more detail below.

MOLECULAR ECOLOGY

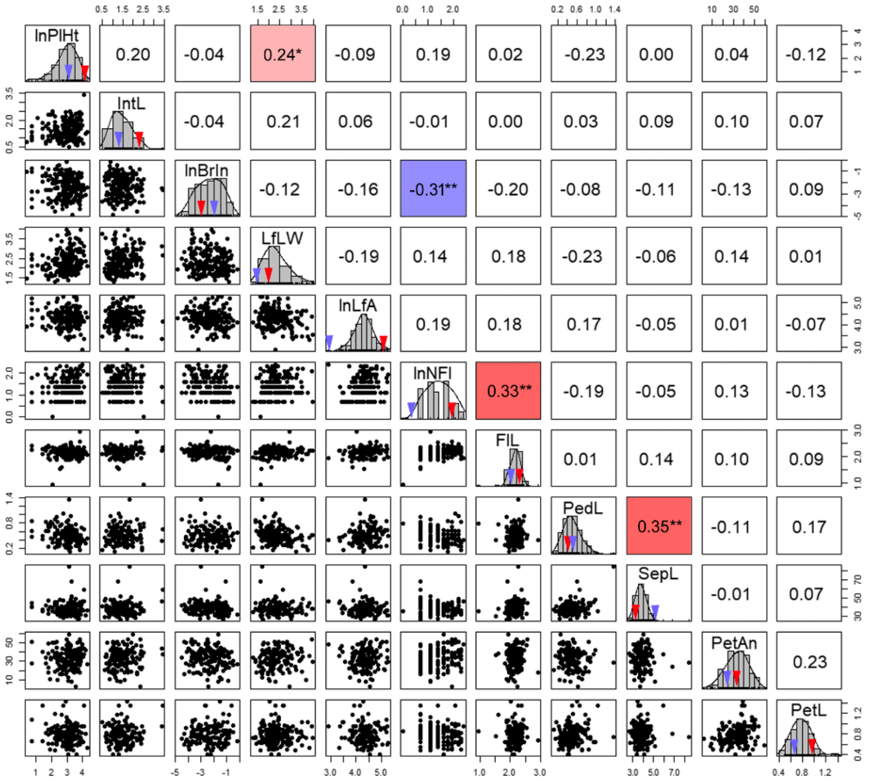

Figure S2 Character variation and correlation in F2 hybrids of *A. rupestre* x *A. barrelieri*

Frequency distributions for each character in the F2 are shown on the diagonal, with the mean values for *A. rupestre* and *A. barrelieri* given by red and blue arrows, respectively. Scatter-plots for pairs of characters are shown below the diagonal. The boxes above give correlation coefficients from least-squares regression with correlated pairs of characters coloured (* $\alpha \leq 0.05$, ** $\alpha \leq 0.01$ after Bonferroni correction).

MOLECULAR ECOLOGY

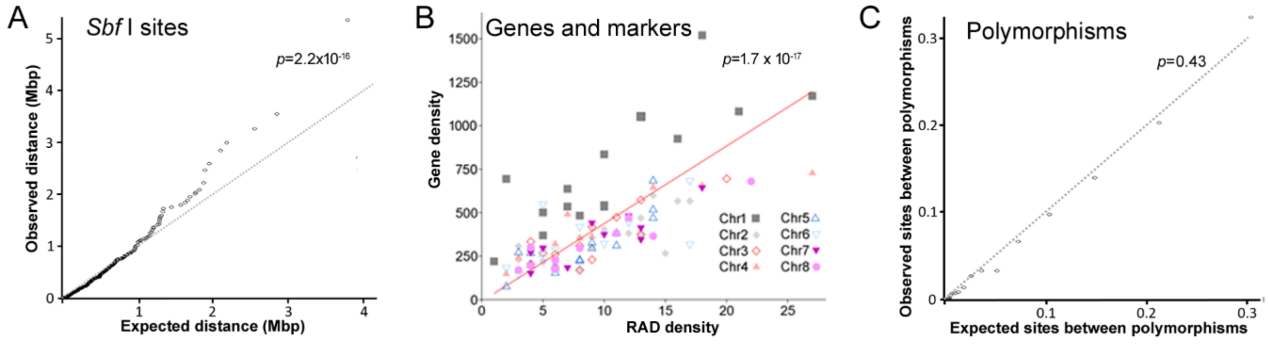

Figure S3 Genome distribution of *Sbf* I sites, genes and RADseq polymorphisms

A) Quantile-quantile plot of physical distances between adjacent *Sbf* I sites in the *A. majus* genome and distances expected for randomly-located loci simulated as a geometric frequency distribution with the same mean (grey line). B) Correlation of *Sbf* I sites with genes in the *A. majus* reference. Density values are for 5-Mbp non-overlapping windows. C) Plot of observed and expected frequencies for the number of fixed RAD-seq loci between each pair of polymorphic loci. *p*-values in A) and C) are from comparisons of observed frequency distributions to the geometric distributions expected for randomly distributed *Sbf* I sites or polymorphisms distributed randomly among RADseq loci, and in B) for gene and RAD densities being uncorrelated.
